# Targeting TNF-α for COVID-19: Recent Advanced and Controversies

**DOI:** 10.3389/fpubh.2022.833967

**Published:** 2022-02-11

**Authors:** Yi Guo, Ke Hu, Yuxuan Li, Chanjun Lu, Ken Ling, Chuanqi Cai, Weici Wang, Dawei Ye

**Affiliations:** ^1^Department of Vascular Surgery, Union Hospital, Tongji Medical College, Huazhong University of Science and Technology, Wuhan, China; ^2^Clinic Center of Human Gene Research, Union Hospital, Tongji Medical College, Huazhong University of Science and Technology, Wuhan, China; ^3^Department of Anesthesiology, Union Hospital, Tongji Medical College, Huazhong University of Science and Technology, Wuhan, China; ^4^Department of Pancreatic-Biliary Surgery, Shanxi Bethune Hospital, Shanxi Academy of Medical Sciences, Tongji Shanxi Hospital, Third Hospital of Shanxi Medical University, Taiyuan, China; ^5^Department of Cancer Center, Tongji Hospital, Tongji Medical College, Huazhong University of Science and Technology, Wuhan, China

**Keywords:** COVID-19, cytokine release syndrome, MIS-C, TNF-α inhibitor, infliximab

## Abstract

Recent advances in the pathophysiologic understanding of coronavirus disease 2019 (COVID-19) suggests that cytokine release syndrome (CRS) has an association with the severity of disease, which is characterized by increased tumor necrosis factor α (TNF-α), interleukin (IL)-6, IL-2, IL-7, and IL-10. Hence, managing CRS has been recommended for rescuing severe COVID-19 patients. TNF-α, one of the pro-inflammatory cytokines commonly upregulated in acute lung injury, triggers CRS and facilitates SARS-CoV-2 interaction with angiotensin-converting enzyme 2 (ACE2). TNF-α inhibitors, therefore, may serve as an effective therapeutic strategy for attenuating disease progression in severe SARS-CoV-2 infection. Below, we review the possibilities and challenges of targeting the TNF-α pathway in COVID-19 treatment.

## Introduction

Severe acute respiratory syndrome coronavirus-2 (SARS-CoV-2), the causative agent of coronavirus disease 2019 (COVID-19), was declared a pandemic by the World Health Organization on 11th March 2020 and has become a major global health concern. According to recent statistical data among all COVID-19 patients, ~19% of them were severe or critical, most of which displayed remarkably increased serum levels of pro-inflammatory cytokines and subsets of immune cells (1–3). This kind of unprecedented spike in cytokines levels in critically ill COVID-19 patients is termed as cytokines release syndrome (CRS). Data obtained from COVID-19 patients have also shown that severe cases may be characterized by a CRS-induced irreversible progression to acute respiratory distress syndrome (ARDS) (4–6). To date, new therapeutic strategies for severe COVID-19 remain limited, and several antiviral drugs like lopinavir/ritonavir have shown no significant improvement in COVID-19 patients compared to those in standard care (7). It is noteworthy that tackling the immune response may be as important as controlling viral replication. Though the vaccines have developed a lot, the uncertainty around global uptake and vaccine safety remain, how to effectively treat patients with COVID-19 still attracts worldwide attention (8).

## The Immune Response and CRS in COVID-19

Recent advances in the pathophysiologic understanding of COVID-19 suggests that CRS has a great association with the severity of disease due to increased serum levels of tumor necrosis factor α (TNF-α), granulocyte-colony stimulating factor (G-CSF), interferon gamma-induced protein 10 (IP-10), monocyte chemoattractant protein 1 (MCP-1) and macrophage inflammatory protein 1α (MIP1A) (4, 6). A study of a longitudinal serum cytokine analysis of 207 COVID-19 patients showed that in very early inflammatory responses, IL-6, TNF-a, IL-10, and IL-1b rose in those with more severe disease (9). Additionally, based on a meta-analysis included about 23 studies, IL-6, IL-8, IL-10, IL-2R, and TNF-α cytokine levels were significantly higher and T-lymphocyte levels were significantly lower among serum of severe cases as compared to non-severe COVID-19 patients (10). Higher expressions of interleukin (IL)-2R, IL-6, IL-10, IL-18, Type-I interferon (IFN)-γ, and TNF-α cytokines in serum seemed to predict the severity and prognosis of SARS-CoV-2 infected patients (11, 12). These cytokines and chemokines were found to be positively associated with SARS-CoV-2 viral load (13). Peripheral blood flow cytometric analysis also indicated that overactivation of T cells accounted for the severe immune injury in this patient to some extent (14). Furthermore, interstitial mononuclear inflammatory infiltrates in both lungs, which are dominated by lymphocytes, were found in the pathological examination of a biopsy sample from a patient who died from COVID-19 (14). The large area of lung injury (≥50%) was closely correlated with the increased level of IL-6 as well as the subgroup of lymphocytes in the peripheral blood, according to the assessment of pulmonary infiltration in patients with ARDS (15). Thus, CRS may serve as an effective target in the treatment of COVID-19.

Initial reports have also determined that a multisystem inflammatory syndrome in children (MIS-C) associated with COVID-19 may cause public concern. It has been reported that patients with MIS-C tend to have variable symptoms such as unrelenting fever, exanthema, conjunctivitis, lymphadenopathy, peripheral oedema, and generalized extremity pain with significant gastrointestinal symptoms. Verdoni et al. (16) found that patients with mild also have a constellation of features that could be classified under the term CRS. It was found that levels of cytokines TNF-α and IL-10 discriminated between patients with MIS-C and severe COVID-19 (17). Another study in France found an elevation of inflammatory parameters including leukocytosis, levels of C-reactive protein (CRP), procalcitonin, and serum IL-6 (18). Moreover, Whittaker et al. found that a group of patients who presented well-respond to a variety of immunomodulatory treatments, including corticosteroids and intravenous immunoglobulin (IVIG), as well as some biologics such as infliximab (IFX), a TNF-α inhibitor, and anakinra, an IL-1 inhibitor (19).

Though Kawasaki disease (KD) and MIS-C share much common ground and similar clinical manifestations, patients with MIS-C were not only generally older than those with KD, but also had a higher white blood cell count, neutrophil count, and CRP (20). They were also inclined to suffer from profound lymphopenia and anemia, as well as lower platelet counts, higher fibrinogen levels, and greater elevation of troponin. This may be related to an overreaction of the body's natural defenses or immune system. It has been hypothesized that some children are predisposed to a more exaggerated inflammatory response to SARS-CoV-2, therefore inducing CRS, resulting in the clinical manifestation of MIS-C.

In summary, all of the aforementioned results suggested that SARS-CoV-2 triggers the aberrant production of cytokines and chemokines as well as alterations in the level of the subgroup of lymphocytes. This immune dysregulation may be responsible for CRS and further tissue damage.

## TNF-α Signaling Pathway in CRS

TNF-α, a major pleiotropic mediator of acute and chronic systemic inflammatory responses, can simultaneously regulate cells apoptosis and proliferation while promoting the production of other chemokines and cytokines. It is also involved in a series of physiological processes such as anti-tumor responses, control inflammation, and immune system homeostasis (21, 22). TNF-α is also one of the most important pro-inflammatory cytokines of the innate immune response, dysregulated TNF-α signaling can trigger CRS (23).

A team studied the immune responses of 54 COVID-19 patients and found that TNF-α and IL-6 production by circulating monocytes was sustained. All their patients with pneumonia caused by SARS-CoV-2 who develop SRF display hyper-inflammatory responses with two features: over-production of pro-inflammatory cytokines by monocytes and dysregulation of lymphocytes characterized by CD4 lymphopenia and subsequently B cell lymphopenia (24). A rise in TNF-α could result in the facilitation of viral infection and organ damage (25). It has been reported that anti-TNF therapy can significantly improve the severe respiratory syncytial virus and influenza in mice, thereby reducing the recruitment of inflammatory cells, disease duration, cytokine production induced by T cells, and the severity of illness (26). Blocking TNF-α can strongly modulate the balance between effector T cells and regulatory T cells; as a result, approved TNF-α inhibitors play a crucial role in some severe autoimmune inflammatory diseases as well as numerous chronic inflammatory diseases such as psoriasis, rheumatoid arthritis (RA), inflammatory bowel disease (IBD) and ankylosing spondylitis, refractory hemophagocytic lymphohistiocytosis, systemic juvenile idiopathic arthritis, Behcet's disease, dermatomyositis, and lupus (27–36). Although TNF coordinates the inflammatory response during the acute phase of inflammation, excessive TNF will suppress the immune system with the development of disease, which may lead to the adverse result (37).

However, inhibiting TNF has a direct effect in reducing the level of IL-6 and IL-1 along with adhesion molecules and vascular endothelial growth factor (VEGF) in RA patients (38). Notably, IL-6, IL-1, adhesion molecules, and VEGF have already been reported in many clinical cohort studies of COVID-19. The plasma level of IL-6, a significant cytokine contributing to macrophage activation syndrome (MAS), which is also known as a form of CRS, has been shown to increase in both mild and severe COVID-19 patients (1, 5, 6, 39). TNF inhibitors have already been successfully used to treat MAS. Significantly, adhesion molecules and VEGF also play a crucial role in pulmonary capillary leak (40–44). TNF can also reduce other inflammatory proteins, such as CRP and serum amyloid A protein (45). TNF-targeting aptamer treatment has also been reported to attenuate the poor prognosis of LPS-induced acute lung injury by improving oxygen saturation and lung injury scores, reducing protein-rich fluid leakage and neutrophil infiltration in alveolar spaces, and suppressing pro-inflammatory cytokines and chemokines expressions in lung tissues (46).

**Figure 1:**
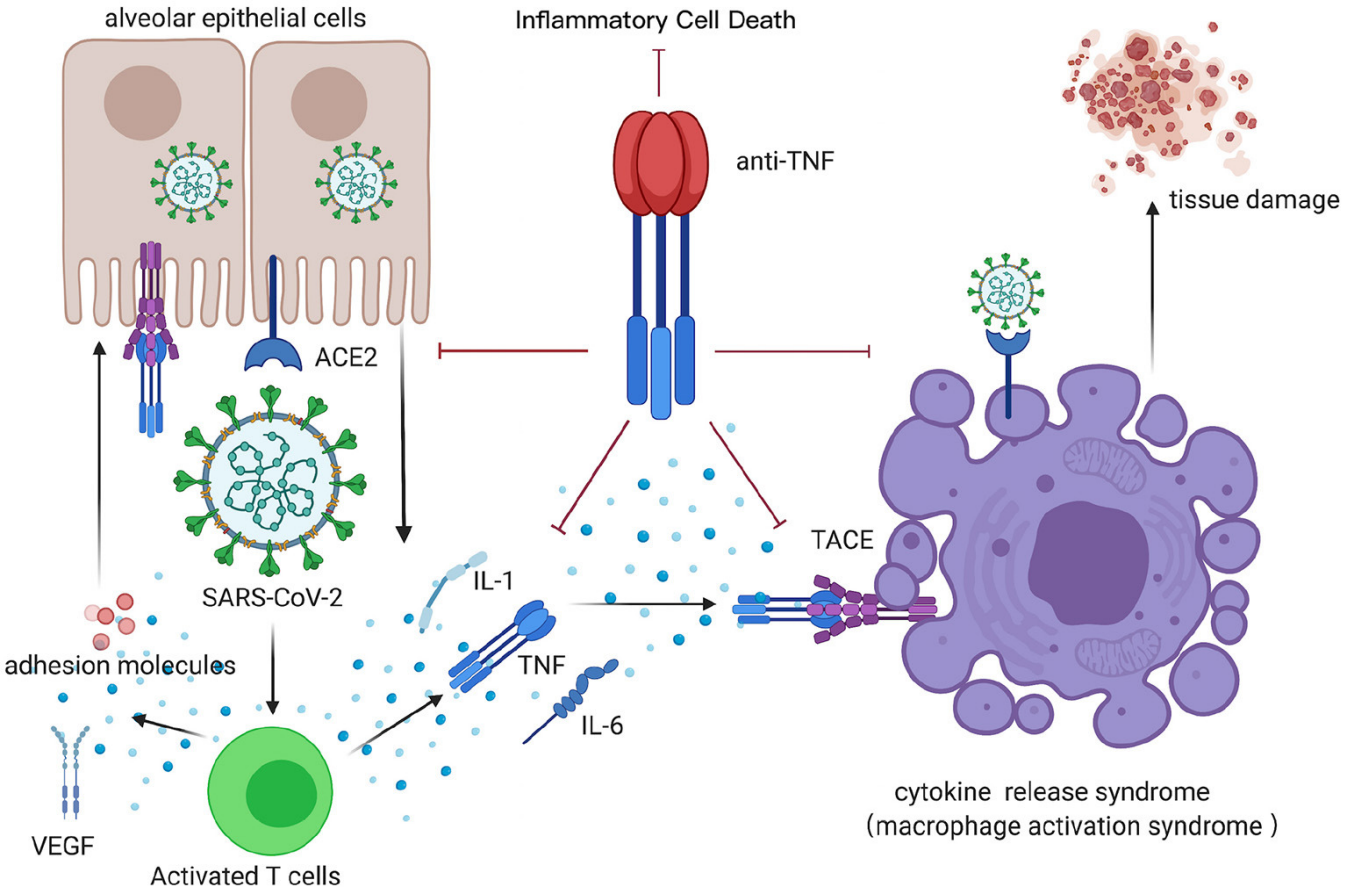
CRS after COVID-19 infection, inhibited by anti-TNF therapy. TNF, tumor necrosis factor; ACE, angiotensin-converting enzyme; TACE, TNF-α-converting enzyme; VEGF, vascular endothelial growth factor; IL-1, interleukin-1; IL-6, interleukin-6. This figure was generated in Biorender (https://Biorender.com).

During SARS-CoV-2 infection, aggressive inflammatory response and cytokine storm contribute to severe systemic tissue damage and mortality. Recently, original research demonstrated that the synergism between TNF-α and IFN-γ is critical in triggering robust cell death during activating JAK/STAT1/IRF1 axis in the human monocytic cell line THP-1 and primary human umbilical vein endothelial cells (12). What's more, the kinetics of cell death induced by TNF-α and IFN-γ co-stimulation were proportional to the concentrations of TNF-α and IFN-γ. Collectively, the TNF-α signaling pathway plays a vital role in CRS (Figure 1). Blocking the cytokine-mediated inflammatory cell death signaling pathway may benefit patients with COVID-19 or other infectious diseases by limiting tissue damage (47).

## Implications on Antibacterial and Antiviral Immunity

According to *in vitro* studies, TNF-α facilitates the SARS-CoV interaction with angiotensin-converting enzyme 2 (ACE2) (48). ACE2, a type I membrane protein detected in many organs, especially lung AT2 alveolar epithelial cells that are particularly susceptible to viral infection, has been established as the host cell-surface receptor for both SARS-CoV and SARS-CoV-2 (49). Though a few studies provided implications that SARS-CoV-2 could accelerate its entrance into the infected cells by leveraging up-regulation of its receptor gene ACE2 via induction of cytokine storm involving IFNG, TNF-α as well as NFKβ (50, 51), physiologically ACE2 still has a significant anti-inflammatory role. The normal level of ACE2 is important to protect vital organs, SARS-CoV-2 infection causes the downregulation of ACE2 expression, leading to an overwhelming production of angiotensin II and resulting in increased pulmonary vascular permeability and lung damage. Loss of ACE2 expression may also increase neutrophil accumulation, lung edema, and diminish lung function (52). Reduced ACE2 activity can indirectly activate the kallikrein-bradykinin pathway and increase vascular permeability (53). Potdar et al. (54) reported that through their univariate analyses, small bowel ACE2 levels were restored after anti-TNF (infliximab) therapy and that it was statistically significant in anti-TNF responders. Moreover, some inflammatory cytokines like IL-1β and TNF-a can strengthen ACE2 shedding (48, 55, 56). The SARS-CoV spike protein can induce a TNF-α-converting enzyme (TACE)-dependent shedding of the ACE2 ectodomain, which may be the crux of the virus penetrating the cell (48). A close correlation between the SARS-CoV spike protein-induced ACE2 shedding and TNF-α production in cell culture conditions have been shown, which may suggest the significance of modulating TNF-α in SARS-CoV (48). SARS-CoV and SARS-CoV-2 share a highly similar mechanism of structures of spike proteins and semblable pathogenicity, and the surface of human ACE2 (hACE2) plays a critical role in both of them, which indicates that modulation of TNF-α may be the crux in SARS-COV-2 as well (57–60).

**Figure 2:**
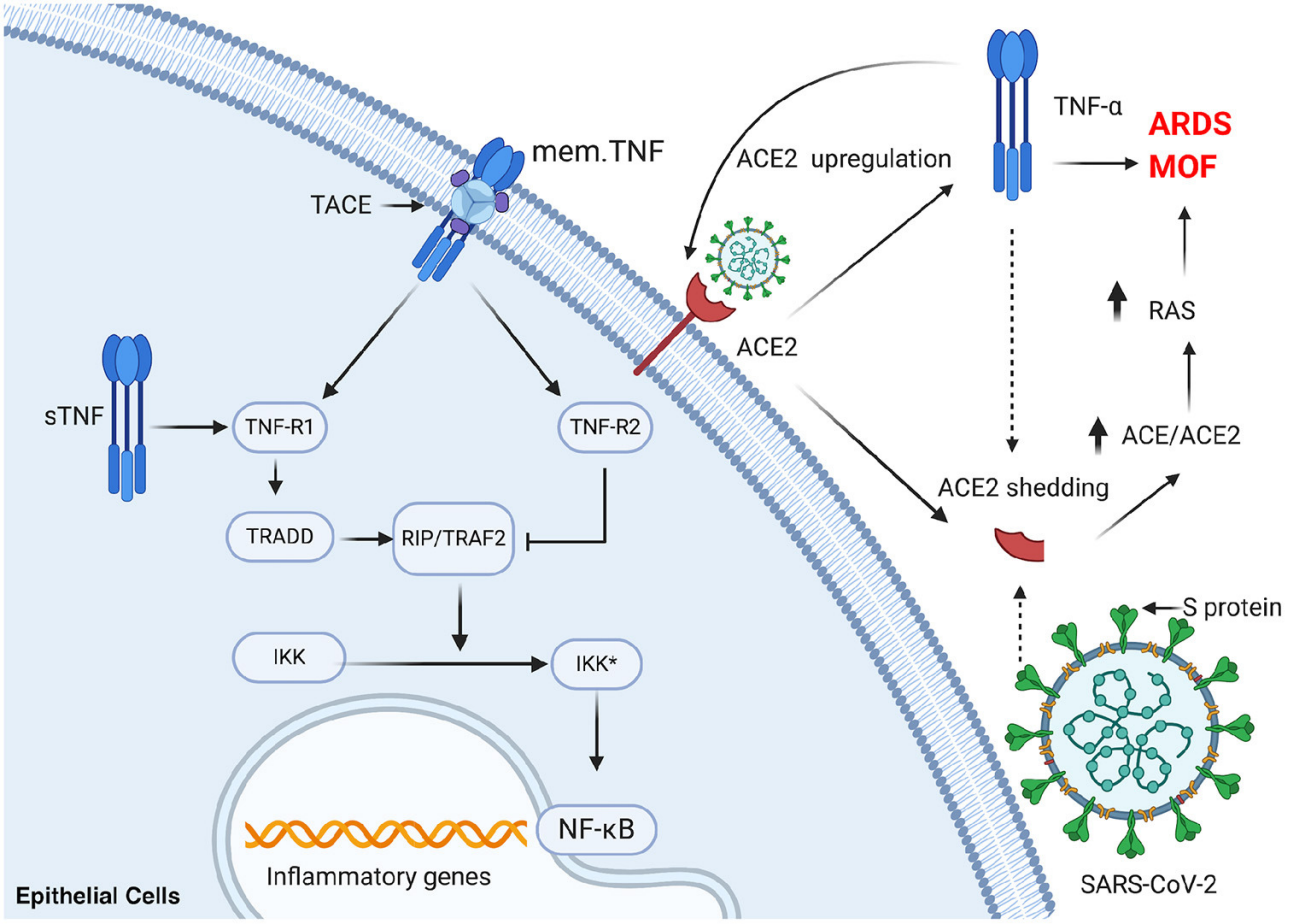
TNF-a plays a significant role in activation of NF-κB and ACE2 shedding in SARS-CoV-2 infection. mem.TNF, membrane-bound TNF; sTNF, soluble TNF; TACE, TNF-α-converting enzyme; TNF-R1, TNF-receptor 1; TNF-R2, TNF-receptor 2; TRADD, TNF-receptor-associated protein; RIP, receptor-interacting protein; TRAF2, TNF receptor-associated factor 2; IKK, I-κB kinase; NF-κB, nuclear factor kappa B; ACE, angiotensin-converting enzyme; ARDS, acute respiratory distress syndrome; MOF, multi-organ failure; RAS, renin-angiotensin system. This figure was generated in Biorender (https://Biorender.com).

Nuclear factor kappa B (NF-κB), a transcription factor modulating both innate and adaptive immunity, is also crucial in the progress of COVID-19 (61). Relevant changes that occur in innate and adaptive immunity in COVID-19 have been highlighted by several studies. Specifically, NF-κB can affect pro-inflammatory cytokines and chemokines, anti-apoptotic proteins, and stress-response proteins by inducing the transcription of a wide spectrum of their encoding genes. NF-κB is also crucial for survival and activation and plays a significant role in initiating and propagating optimal immune responses (62). A study is reported that an exacerbated inflammatory response in severe COVID-19 patients associated with increased TNF-a and IL-6 potentially driven by NF-κB (63). Notably, respiratory viruses including SARS-CoV can induce the exacerbated activation of NF-κB, leading to lung inflammatory immunopathology (64, 65). Additionally, blocking NF-κB can protect against pulmonary pathology and enhance mice survival after SARS-CoV infection (64). All of the aforementioned evidence suggests that blocking rate NF-κB may be a consequential choice in counteracting disease progress in COVID-19 patients. However, blocking NF-κB may bring about undesirable side effects, such as the broad suppression of innate immunity due to the lack of specificity of the relevant treatment and the significance of NF-κB in cellular homeostasis (66). The interaction of TNF receptor-associated factor (TRAF) 2 with the I-kB kinase (IKK) complex, as well as the support of receptor-interacting protein (RIP), can finally activate NF-κB (67). TRAF 2, a vital constituent of the TNF receptor signaling complexes, participates in the TNF-R1-mediated activation of NF-kB (68). As a result, blocking TNF-α may be a more effective strategy, which have been clinically and effectually used in several pathological conditions (Figure 2).

## Potential of TNF-α Inhibitors in COVID-19

Since TNF-α production plays a vital role in these processes, inhibiting TNF-α may be an effective strategy in attenuating both SARS-CoV2 infection and the resulting organ damage (69). Feldmann et al. suggested to evaluate anti-TNF therapy as soon as COVID-19 patients are admitted to the hospital to prevent their condition from persisting or worsening, which would then require intensive care support. It may be reasonable to initiate anti-TNFα therapy as early as possible (70). Existing anti-TNF therapies include Humira (adalimumab, AbbVie), Remicade (infliximab, Janssen), Simponi (golimumab, Janssen), Cimzia (certolizumab pegol, UCB), and Entyvio (vedolizumab, Takeda). Currently, a clinical trial comprised of COVID-19 patients evaluating adalimumab, an anti-TNF-α drug, has been registered in China (ChiCTR2000030089). A phase II Trial of IFX in COVID-19 registered in the US (NCT04425538) has been completed and reported that IFX may abrogate pathological inflammatory signaling rapidly to facilitate clinical recovery in severe and critical COVID-19 (71). Additionally, a prospective multicenter cohort of patients with IBD Treated by IFX or Vedolizumab during the COVID-19 pandemic was registered in the US (NCT04344249). A few studies have reported recovery in a patient with COVID-19 treated with TNF-α inhibitor etanercept successfully (32, 34, 72, 73). Several trials with different TNF antagonists will provide information on the effectiveness of these therapeutic interventions along with the risks of these measures in COVID-19 therapy, are shown in Table 1.

**Table 1:** Drugs targeting TNF or its receptor in clinical studies in patients with COVID-19.

**Drug treatment**	**Mode of action**	**Patient category**	**Primary endpoint**	**Estimated**	**Trial/phase (as**	**Testing status**
				**enrollment**	**of 07/18/2021)**	
XPro1595	Soluble TNF inhibitor	COVID-19 with pulmonary complications	The proportion of participants who die or require mechanical ventilation.	366	NCT04370236, phase 2/3	Recruiting
CERC-002	LIGHT(TNFSF14) inhibitor	COVID-19 with mild to moderate ARDS	The proportion of patient alive and free of respiratory failure	82	NCT04412057, phase 2	Completed
Infliximab	Chimeric monoclonal anti-TNF antibody	COVID-19	Time to improvement in oxygenation	17	NCT04425538, phase 2	Completed
	Chimeric monoclonal anti-TNF antibody	COVID-19	28-day mortality	88	NCT04922827, phase 2	Recruiting
Tocilizumab /Infliximab	TNFα inhibitor (Infliximab)	COVID-19-associated Cytokine Storm Syndrome	Time to improvement in oxygenation	84	NCT04734678	Recruiting
Adamumab (Qletli)	TNFα inhibitor	Severe and critical COVID-19	Time to clinical improvement	30	ChiCTR2000030089	Not yet recruiting
Infliximab/vedolizumab	TNFα inhibitor (infliximab)	IBD and COVID-19	IgG and IgM anti SARS-CoV-2	850	NCT04344249	Recruiting

IFX, a recombinant humanized monoclonal antibody against TNF-α, is now approved for use in several immune-mediated disorders, including psoriasis, RA, IBD, ankylosing spondylitis, and IVIG resistant KD (74–77). Numerous studies have reported that combined treatment with IFX was related to clinical and biochemical improvement in KD complicated with MAS (78). Considering the potential similar pathophysiology between KD complicated with MAS and MIS-C, which is also potentially related to CRS to a certain extent, IFX may play a vital role in CRS with COVID-19. Additionally, IFX has already been reported in successfully treating patients with MIS-C when treating the COVID-19 inflammatory cascade (79). IFX has also demonstrated collateral benefits for an adult patient diagnosed with severe ulcerative colitis and COVID-19 as both diseases successfully improved (80).

A coronavirus and IBD reporting database on Surveillance Epidemiology of Coronavirus Under Research Exclusion (SECURE-IBD) has shown a cluster of outcomes for IBD patients diagnosed with COVID-19. As of December 7, 2021, 2216 patients have been reported to use anti-TNF therapy alone, of which 2004 patients were rehabilitated without hospitalization, while 10 died with a mortality rate of <1%. Meanwhile, 25 required transfer to the ICU, of which 18 required a ventilator. Together, the rate of patients with poor prognosis involving ICU transfer, ventilation or death was 1%. However, only 1,593 of 2,035 patients who used sulfasalazine/mesalamine recovered without hospitalization, while 53 died with a mortality rate of 3%. Here, 82 were transferred to the ICU, of which 70 needed ventilation. The rate of those using sulfasalazine/mesalamine with a poor prognosis was about 6%. Moreover, anti-TNF therapy maintained the highest outpatient rate of 90% among all kinds of IBD medications in the database, even compared to the IL 12/23 inhibitor and JAK inhibitor. Research based on the SECURE-IBD database also suggests that TNF antagonist treatment was not an independent risk factor for severe COVID-19 and plays a significant protective role (81, 82). Anti-TNF therapy was found to be related to reduced probability of hospitalization while prednisone use ≥10 mg/day was linked with a higher probability of hospitalization (83). Evidently, thiopurine monotherapy and the combination thiopurines with TNF antagonists are associated with significantly increased risk of severe COVID-19 compared with TNF monotherapy. Therefore, consideration of discontinuing the thiopurine during the COVID-19 pandemic may be warranted in those high-risk patients (e.g., older age or multiple comorbidities) in stable remission on TNF antagonist combination therapy (84).

Furthermore, there have been many case reports of patients using TNF inhibitors as treatment for COVID-19, demonstrating no respiratory complications or death (72, 85, 86). More significantly, Bezzio and colleagues found no association between the use of biological therapies and negative outcomes of COVID-19 in IBD patients (87). In a study of 70 rheumatic disease patients, patients on anti-TNF alpha medications were hospitalized less frequently (88). Another study reported that COVID-19 prevalence in patients with IBD was comparable with that in the general population and anti-TNF agents seem to mitigate the course of COVID-19 (89). According to a case-control study which investigated the frequency of COVID-19 incidence in 254 eligible patients with rheumatoid arthritis (RA) or seronegative spondyloarthropathies (SpA), TNF-α blockers including adalimumab, infliximab, and etanercept decreased significantly the risk of developing COVID-19 in patients with RA and SpA. And the highest percentage of impact on the prevention of COVID-19 was related to adalimumab (96.8%). Their result could also confirm that TNF-α inhibitors may mitigate or ameliorate the COVID-19 disease course (90). Overall, patients with COVID-19 using anti-TNF therapy do not fare worse than those treated with other drugs.

## Controversy on the Safety of Anti-TNF Therapy in Patients With COVID-19

TNF-α inhibitors may trigger or worsen disease progression (33). TNF-α inhibitors can increase bacterial and mycobacterial infections in patients as well as reactivate latent infections, including hepatitis B and tuberculosis, with its immunosuppressive activity (91). A systematic review and meta-analysis of published trials found a higher incidence rate of any infection (20%), serious infection (40%), and tuberculosis (250%) related to anti-TNF therapy (92). Moreover, patients already taking immunosuppressive drugs such as TNF inhibitors are potentially an at-risk group for COVID-19 infection and its complications (93, 94). Meanwhile, paradoxical effects such as sarcoidosis and other granulomatous disorders like rheumatoid nodules and vasculitis, paradoxical psoriasis and psoriasiform lesions, uveitis and other eye diseases, and miscellaneous auto-immune diseases like central and peripheral nervous system demyelinating disorders, have also been reported to be associated with anti-TNF drug use (95). However, most symptoms could resolve after the discontinuation of anti-TNF therapy or switching to another anti-TNF agent.

As a result, it is of great importance to differentiate patient cohorts with a bleak prognosis and amendable clinical outcomes who require anti-TNF therapy from those who can be rehabilitated on their own to minimize potential risks linked with immunosuppression. Prophylactic treatment and screening for latent tuberculosis while assessing the risk of venous thromboembolism prior to commencing anti-TNF therapy are still highly recommended.

IFX exposure was found to be independent of increased risk of hemophagocytic lymphohistiocytosis in pediatric patients with IBD who were followed for 20 years in a prospective cohort study (96). A study conducted comprised of 434 pediatric patients with KD received IFX in Japan, which also showed no MAS cases have been reported. In this study, the median days of IFX treatment were 9, and single dose IFX 5 mg/kg was administered to 412 patients (94.9%) (76). As an approved drug for pediatric patients with low risk of MAS occurrence, IFX may serve as a proper choice among all TNF inhibitors. However, although using IFX (5 mg/kg) against CRS with organ failure in COVID-19 patients 3 days has shown temporary reductions in pro-inflammatory cytokines, it may increase the risk of concomitant infection according to an analysis of a cautionary case series (97).

## Conclusions and Future Perspectives

All of the corresponding results suggest the security and potential significance of TNF inhibitors to a certain degree. Thus far, anti-TNF therapy seems to have had a protective effect on the progression of COVID-19, especially in severe forms. In addition, anti-TNF therapy has a well-demonstrated ability to reduce inflammation and can prevent CRS associated with the immune pathogenesis of infection. Research based on the SECURE-IBD database has shown that TNF antagonist treatment reduces the ratio of COVID-19 patients with a poor prognosis and plays a significant protective role. However, there are still some limitations, such as the data from the SECURE-IBD database, anti-TNF drugs are only used in COVID-19 patients with IBD, not including non-IBD COVID-19 patients. Nevertheless, some other clinical trial results suggest that anti-TNF therapy is meaningful in COVID-19 patients. The currently completed clinical trials are mainly aimed at IFX, which has been proved to facilitate clinical recovery in severe and critical COVID-19. The clinical trial on adalimumab also has been recruited, and the role of the TNF-inhibitor family in covid-19 patients is appreciable. Notably, the adoption of anti-TNF therapy may be limited by its potential side effects, which underlines the significance of differentiating patient cohorts with a bleak prognosis and amendable clinical outcomes from those who can be rehabilitated on their own, as discussed earlier. Finding the timing of the therapy in COVID-19 patients also appears to be crucial. Therefore, we're looking forward to the results of clinical trials to bring us more guiding advice. In a word, the role of TNF inhibitors in the treatment of COVID-19 warrants further study.

## Author Contributions

YG, KH, and YL: served as a guarantor, contributed to literature search, manuscript editing, manuscript preparation, interpretation of data, and study design. CL: contributed to design, interpretation of data, literature search, and manuscript preparation. KL and CC: contributed to manuscript preparation, interpretation of data, and manuscript review. WW and DY: served as a guarantor, contributed to literature search, manuscript review, manuscript editing, manuscript preparation, interpretation of data, and study design. All authors read and approved the final version to be published.

## Funding

This review was supported by funding from the National Natural Science Foundation of China (Grant Numbers 82000729 and 81873529) and Shanxi Province 136 Revitalization Medical Project Construction Fund.

## Conflict of Interest

The authors declare that the research was conducted in the absence of any commercial or financial relationships that could be construed as a potential conflict of interest.

## Publisher's Note

All claims expressed in this article are solely those of the authors and do not necessarily represent those of their affiliated organizations, or those of the publisher, the editors and the reviewers. Any product that may be evaluated in this article, or claim that may be made by its manufacturer, is not guaranteed or endorsed by the publisher.
